# Xanthogranulomatous Inflammatory Pelvic Mass Mimicking Malignancy: Successful Conservative Treatment and Narrative Insights into Diagnosis and Management

**DOI:** 10.3390/jcm15114066

**Published:** 2026-05-25

**Authors:** Carmine Siniscalchi, Augusto Vaglio, Alessandro Palumbo, Beatrice Prati, Antonio Nouvenne, Alberto Parise, Nicoletta Cerundolo, Domenico Corradi, Jean-Francois Emile, Claudio Tana, Tiziana Meschi

**Affiliations:** 1 Internal Medicine Department, Parma University Hospital, 43125 Parma, Italy; csiniscalchi84@gmail.com (C.S.); antonio.nouvenne@unipr.it (A.N.); aparise@ao.pr.it (A.P.); ncerundolo@ao.pr.it (N.C.); tiziana.meschi@unipr.it (T.M.); 2 Department of Biomedical, Experimental and Clinical Sciences, University of Florence, 50121 Florence, Italy; augusto.vaglio@unifi.it; 3 Nephrology and Dialysis Unit, Meyer Children’s Hospital IRCCS, 50139 Florence, Italy; 4 Radiology Department, Parma University Hospital, Via Gramsci 14, 43125 Parma, Italy; alepalumbo@gmail.com; 5 Parma University Hospital, 43125 Parma, Italy; domenico.corradi@unipr.it; 6 Université Paris-Saclay, Université de Versailles Saint-Quentin-en-Yvelines (UVSQ), Inserm U981, 91190 Boulogne-Billancourt, France; jean-francois.emile@uvsq.fr; 7 AP-HP, Hôpital Ambroise-Paré, Service de Pathologie, Smart Imaging Platform, 92100 Boulogne-Billancourt, France; 8 Internal Medicine Unit, Eastern Hospital of Taranto, 74024 Taranto, Italy; claudio.tana@yahoo.it

**Keywords:** xanthogranulomatous inflammation, pelvic mass, histiocytic disorders, mTOR inhibitors, everolimus, conservative management, IgG4-related disease

## Abstract

Pelvic xanthogranulomatous inflammation is a rare pathological entity that can closely mimic malignant disease on cross-sectional imaging, often leading to consideration of radical surgical intervention. We report the case of a 59-year-old woman who presented with a large retrovesical pelvic mass initially suspected to be a malignant process. A definitive diagnosis was established only after tissue biopsy and comprehensive histopathological examination, which excluded malignancy and demonstrated xanthogranulomatous histiocytic inflammation. In light of the lesion’s anatomical location and the substantial morbidity associated with surgical resection, a conservative medical strategy was pursued. Treatment with systemic corticosteroids and everolimus led to marked clinical improvement and a substantial radiological response, with reduction in lesion size from 41 × 26 mm to 27 × 17 mm, thereby allowing avoidance of mutilating surgery. This case underscores the critical role of biopsy and expert pathological assessment in guiding clinical decision-making and supports the consideration of non-surgical therapeutic approaches in selected patients with xanthogranulomatous pelvic lesions.

## 1. Introduction

Xanthogranulomatous (XG) inflammation is an uncommon, destructive form of chronic inflammation that can involve a wide range of abdominal and pelvic organs and often manifests as a mass-like lesion on imaging, sometimes associated with necrosis, cystic change, adhesions, or apparent invasion of adjacent structures. Owing to the non-specific nature of these radiological features, XG inflammation is frequently misinterpreted as a malignant disease, particularly when it arises in anatomically complex regions such as the retrovesical space or the retroperitoneum [1,2]. In both radiological and pathological series, XG processes have been described along a spectrum ranging from infectious–inflammatory conditions to proliferative histiocytic disorders, underscoring the importance of integrating imaging, histopathology, and clinical findings for an accurate diagnosis [1,2]. When occurring in the pelvis, xanthogranulomatous lesions may closely resemble malignancy, presenting as infiltrative masses with heterogeneous enhancement and apparent extension into adjacent tissues; consequently, imaging alone may prompt extensive oncological investigations despite the benign inflammatory nature of the underlying process [1,2].

A further diagnostic challenge arises from the histological overlap between reactive xanthogranulomatous inflammation and non-Langerhans cell histiocytoses, particularly Erdheim-Chester disease (ECD) [3]. ECD is a clonal histiocytic neoplasm characterized by foamy CD68-positive, CD1a-negative histiocytes within a fibro-inflammatory background, typically associated with multisystem involvement and recurrent somatic mutations in genes involved in the MAPK-pathway, namely *BRAF*^V600E^ [4,5]. Current evidence indicates that the diagnosis of ECD should be based not solely on histological and immunophenotypic findings, but on their concordance with clinical and radiological features and, when available, molecular confirmation [6,7].

Among these, IgG4-related disease represents one of the most important non-neoplastic mimickers in the retroperitoneal and pelvic regions, further complicating the diagnostic framework.

In this context, beyond reporting a single clinical observation, this work aims to provide a focused narrative perspective on xanthogranulomatous inflammation, emphasizing its underlying pathophysiology, major imaging pitfalls, and current management strategies. Particular attention is given to the potential role of conservative medical approaches in anatomically complex cases, where surgical intervention may be associated with substantial morbidity.

## 2. Case Presentation and Clinical Context

A 59-year-old woman with a history of rectal adenocarcinoma, treated in 2010 with neoadjuvant chemoradiotherapy followed by low anterior resection and permanent ileostomy, presented with new-onset pelvic symptoms after a prolonged period of clinical stability. Long-term oncological follow-up had revealed no evidence of disease recurrence.

From late 2024, she complained of progressively worsening burning pelvic pain associated with lower urinary tract symptoms, including dysuria and a sensation of pelvic pressure, together with increasing fatigue. The pain involved the suprapubic and perineal regions and was exacerbated by sitting and ambulation. On physical examination, the patient appeared in mild distress. The right iliac ileostomy was functioning normally. Cardiopulmonary examination was unremarkable, and abdominal examination disclosed mild suprapubic tenderness without guarding or rebound. Vital signs were within normal limits, and electrocardiography showed sinus rhythm.

Initial laboratory investigations were largely unremarkable, with normal renal and hepatic function, complete blood count, and coagulation profile. Inflammatory markers, including C-reactive protein and procalcitonin, were within the reference range, whereas the erythrocyte sedimentation rate was mildly elevated (34 mm/h). Tumor markers (CEA, CA-125, CA19-9, and CA15-3) were all within normal limits. Urine culture grew *Staphylococcus aureus*. Abdominal and pelvic ultrasonography did not reveal a definite cause for the patient’s symptoms. No additional microbiological evidence of systemic infection was identified, and blood cultures were negative.

## 3. Diagnostic Work-Up and Imaging Findings

Given the persistence of pelvic pain and urinary symptoms, cystoscopic evaluation was performed (early April 2025) and revealed abnormalities involving the bladder base and retro trigonal region, characterized by fibrin-covered areas and inflamed-appearing mucosa, raising suspicion of contiguous inflammatory involvement or extension from an adjacent pelvic process.

**Figure 1:**
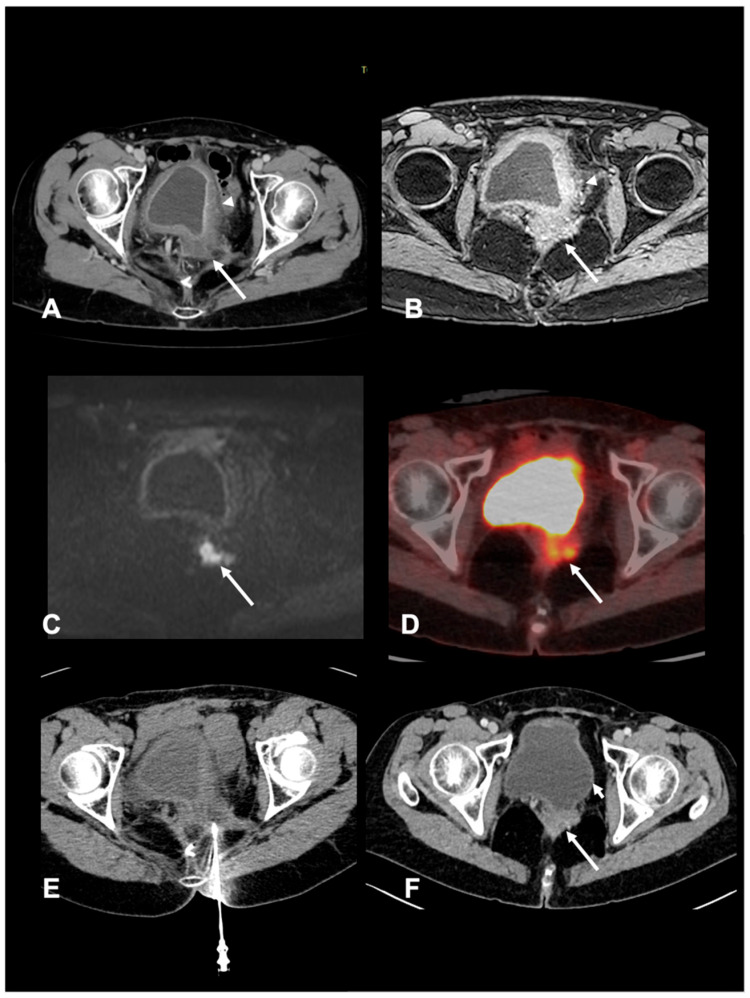
Multimodal imaging, image-guided biopsy, and radiological response after medical therapy of rectovesical xanthogranulomatous inflammatory mass. Panel legend. (**A**) CT of the pelvis demonstrated parietal thickening of the left wall (arrowhead) and posterior wall (arrow) of the bladder with a solid mass approximately 41 × 26 mm. The edges of the tissue were blurred and a peripheral rim enhancement was observed. (**B**) MRI of the pelvis confirmed and better depicted the pathological tissue (arrow), with more evident contrast enhancement compared with CT. Diffusion-weighted imaging (DWI) evidenced high cellularity (arrow). (**C**) Diffusion-weighted imaging (DWI) evidenced high cellularity (arrow). (**D**) PET-CT highlighted FDG uptake of the nodular portion of the pathological tissue, with very good agreement with DWI findings (arrow). (**E**) CT-guided biopsy with an 18G cutting needle of the nodular component of the lesion (arrow). (**F**) Follow-up CT after 6 months of therapy demonstrated a marked reduction in pathological tissue size, with normalization of the left bladder wall (arrowhead) and shrinkage of the posterior nodular component to approximately 27 × 17 mm (arrow).

Contrast-enhanced computed tomography (CT) of the abdomen and pelvis demonstrated a posterior perivesical/retrovesical mass measuring approximately 41 × 26 mm, interpreted as compatible with a chronic inflammatory process, abscess formation, or infiltrative disease (Figure 1A). In the setting of recurrent urinary tract infections, differential diagnoses included chronic abscess or phlegmon and urachal-related inflammatory disease; however, a malignant process could not be excluded on imaging alone. Pelvic contrast-enhanced magnetic resonance imaging (MRI) performed in April 2025 further characterized the lesion as a solid rectovesical mass of approximately 4 cm, with enhancement patterns suggestive of an aggressive soft-tissue lesion (Figure 1B). Diffusion-weighted imaging (DWI) demonstrated areas of high signal intensity consistent with increased cellularity (Figure 1B,C), and PET–CT showed intense FDG uptake corresponding to the nodular component of the lesion (Figure 1D).

Retrospective review of prior imaging revealed that a pelvic MRI performed in 2018 had already demonstrated a hypointense parietal band involving the bladder wall, which had been interpreted at the time as post-treatment fibrosis, while a contemporaneous PET–CT scan showed no evidence of metabolically active disease. Together, these findings were consistent with a long-standing, indolent process.

**Figure 2:**
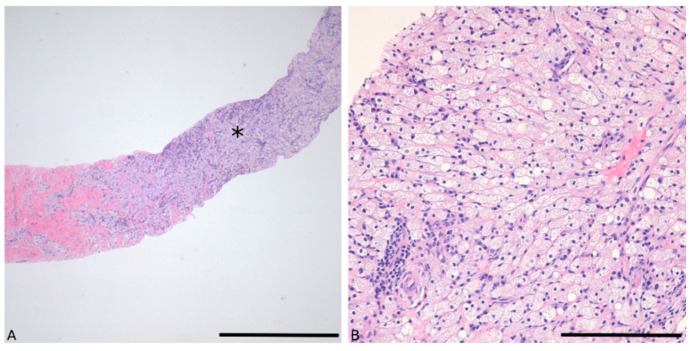
Histopathological features of the rectovesical xanthogranulomatous inflammatory lesion. Scale bars: 1 mm in panel (**A**) and 400 μm in panel (**B**). The asterisk marks the xanthogranulomatous histiocytic infiltrate. (**A**). Low-power view of the needle biopsy showing a significant histiocyte infiltration (asterisk) in a fibrous background. (**B**). At a higher magnification, these histiocytes, without atypia, display a foamy cytoplasm. Foci of chronic inflammation are detectable. Staining: (**A**,**B**), hematoxylin-eosin. Magnification: (**A**) image captured through a ×4 objective (bar is 1 mm), (**B**) image captured through a ×10 objective (bar is 400 microns).

In view of these findings, a CT-guided core needle biopsy of the nodular component of the lesion was performed to obtain tissue for definitive diagnosis (Figure 1E). Histopathological examination showed a proliferation of foamy histiocytes within a chronic inflammatory background, consistent with xanthogranulomatous inflammation, with no evidence of carcinoma (Figure 2A,B). The differential diagnosis included Erdheim-Chester disease and IgG4-related disease. The absence of BRAFV600E mutation, lack of systemic involvement, and the localized nature of the lesion favored a reactive xanthogranulomatous process rather than a clonal histiocytic disorder. At higher magnification, the histiocytes displayed abundant foamy cytoplasm without cytological atypia, accompanied by focal chronic inflammatory infiltrates. Immunohistochemistry demonstrated positivity for CD68 and CD163, with negative staining for S100. Molecular analysis using high-sensitivity digital PCR did not detect the *BRAF*^V600E^ mutation, while focal low-level pERK expression was observed in a subset of histiocytes. The detection of focal pERK expression in a subset of histiocytes suggests activation of the MAPK signaling pathway. Given the known crosstalk between MAPK and PI3K/mTOR pathways in histiocytic disorders, this finding may provide a biological rationale for the use of mTOR inhibition in this case.

The histological findings were independently reviewed at two referral centers and further discussed within a dedicated histiocytosis network, including specialist consultation.

Given the deep pelvic location of the lesion and its close anatomical relationship with the bladder base, surgical resection was considered inappropriate, as complete excision would have required radical cystectomy with urinary diversion, entailing substantial morbidity in the absence of proven malignancy. A conservative medical approach was therefore adopted.

The patient was treated with an anti-inflammatory and immunomodulatory regimen consisting of oral prednisone (25 mg daily for one month, followed by gradual tapered to a maintenance dose of 5–7.5 mg/day), and everolimus initiated at 1 mg twice daily. The everolimus dose was subsequently adjusted to 1.5 mg twice daily, with therapeutic drug monitoring maintaining plasma concentrations within the range of 5–7 ng/mL. Treatment was well tolerated, without clinically significant adverse events.

Clinical improvement was observed within a few weeks, with progressive reduction in pelvic pain and urinary symptoms. At follow-up, the patient reported sustained symptomatic benefit, with only occasional mild discomfort and minimal need for analgesic therapy. A follow-up contrast-enhanced CT scan performed in November 2025 demonstrated a marked reduction in lesion size from 41 × 26 mm at baseline to 27 × 17 mm at follow-up. At the most recent assessment, the patient was asymptomatic, and conservative medical management was continued.

## 4. Differential Diagnosis

The differential diagnosis was progressively refined as imaging and histopathological data became available. At initial presentation, a pelvic malignancy was considered the leading hypothesis, including urothelial carcinoma or metastatic recurrence of the patient’s prior colorectal cancer.

In parallel, an infectious or inflammatory mass was also contemplated, such as a chronic abscess or phlegmon related to recurrent urinary tract infections. Inflammatory pseudotumor, including inflammatory myofibroblastic lesions, was likewise included in the differential diagnosis, given its propensity to present as a bladder-associated mass with radiological features that can closely mimic neoplasia.

Fibrosing inflammatory disorders of the retroperitoneum, particularly IgG4-related disease (IgG4-RD), represent a crucial differential diagnosis in this setting. IgG4-RD is a systemic fibroinflammatory condition characterized by tumefactive lesions, dense lymphoplasmacytic infiltrates, storiform fibrosis, and, in many cases, elevated serum IgG4 levels. Immunohistochemical staining for IgG4 was performed and did not reveal a significant increase in IgG4-positive plasma cells. Quantitative data regarding the absolute number of IgG4-positive plasma cells and the IgG4/IgG ratio were not systematically recorded and were not available for retrospective analysis. However, serum IgG4 levels were within the normal range, and no characteristic histopathological features of IgG4-related disease, such as storiform fibrosis or obliterative phlebitis, were identified. Taken together, these findings were considered insufficient to support a diagnosis of IgG4-related disease.

Importantly, IgG4-RD frequently involves the retroperitoneum and pelvic compartments and may closely mimic malignant disease both clinically and radiologically, presenting as infiltrative masses with apparent extension to adjacent structures [8].

From a clinical perspective, IgG4-RD is particularly relevant because it typically shows a marked response to corticosteroid therapy, which may further overlap with the behavior of other inflammatory conditions. Therefore, failure to consider this diagnosis may lead to unnecessary surgical interventions.

In the present case, IgG4-RD was carefully considered; however, the absence of key histopathological features, such as a dense IgG4-positive plasma cell infiltrate and storiform fibrosis, together with the lack of systemic involvement, made this diagnosis unlikely. These findings supported the interpretation of a localized xanthogranulomatous inflammatory process.

Finally, following the identification of CD68-positive, CD1a-negative foamy histiocytes on histological examination, ECD was taken into account because of its characteristic histiocytic infiltrate within a fibrotic inflammatory background. However, in the absence of multisystem involvement and given the localized nature of the pelvic lesion, a reactive xanthogranulomatous process was considered the more likely diagnosis, pending comprehensive clinicopathological correlation [4,5,6,7,8,9,10,11,12].

## 5. Therapeutic Strategy and Clinical Course

The decision to pursue systemic therapy was primarily driven by the need to avoid mutilating surgery and was further supported by therapeutic principles derived from the management of histiocytic disorders, particularly Erdheim-Chester disease (ECD). mTOR inhibition has previously demonstrated clinical activity in patients with ECD, including cases refractory to conventional therapies, supporting the rationale for targeting downstream inflammatory and proliferative signaling pathways in histiocytic-driven conditions [13,14,15,16]. Although the present lesion did not meet criteria for ECD, the shared histiocytic and inflammatory background provided a biologically plausible basis for adjunctive mTOR inhibition in combination with corticosteroids.

From a management perspective, the present case is consistent with emerging observations suggesting that conservative treatment strategies may be considered in selected cases. While surgical resection has traditionally been pursued due to diagnostic uncertainty, this approach may be associated with considerable morbidity, particularly in anatomically complex regions such as the pelvis. In this context, the combination of corticosteroids and mTOR inhibition represents a biologically plausible strategy targeting both inflammatory and proliferative pathways involved in histiocytic-driven processes. However, evidence remains limited, and further studies are required to validate this approach.

## 6. Discussion: Pathophysiology, Imaging Pitfalls, and Management Considerations

### 6.1. Case-Specific Considerations

This case exemplifies a complex diagnostic and therapeutic scenario in which a rectovesical pelvic mass in a patient with a previous history of malignancy raised strong suspicion for an aggressive or neoplastic process. In this patient, several predisposing factors may have contributed to the development of this condition, including prior pelvic radiotherapy, surgical manipulation, and recurrent urinary tract infections, particularly with *Staphylococcus aureus*. More broadly, xanthogranulomatous (XG) inflammation represents a well-recognized diagnostic pitfall, as it can closely mimic malignancy due to its mass-forming behavior, infiltrative appearance, and heterogeneous imaging characteristics. From a pathophysiological perspective, XG inflammation is characterized by the accumulation of lipid-laden macrophages within a chronic inflammatory and fibrotic background, often associated with tissue destruction and local extension.

In pictorial reviews of abdominal and pelvic XG disease, such lesions are repeatedly described as “malignancy mimics”, owing to their irregular margins, variable contrast enhancement, and apparent involvement of surrounding compartments. Likewise, broader radiological reviews of xanthogranulomatous processes in the abdomen and pelvis emphasize that imaging findings are inherently non-specific and may closely simulate aggressive neoplasms, making histological confirmation essential [1,2].

Within the pelvis, xanthogranulomatous inflammation has been reported in association with both urinary bladder pathology and the female genital tract [10,11]. Case reports and small series describe presentations characterized by mass-like lesions, pelvic pain, urinary symptoms, and imaging findings highly suspicious for malignancy [12]. Xanthogranulomatous cystitis, although rare, is a recognized imitator of bladder cancer, with PubMed-indexed reports describing enhancing bladder-associated masses with suspected local invasion that often prompt partial cystectomy or extensive oncological work-up because malignancy cannot be reliably excluded preoperatively [10,11,12]. Similarly, urachal xanthogranulomatous inflammation has been described as mimicking urachal carcinoma on CT, further illustrating how deep midline pelvic lesions are diagnostically challenging [10,11,12]. These observations closely mirror the clinical course in our patient, in whom malignancy and invasive inflammatory disease remained leading considerations until biopsy clarified the underlying process.

A major diagnostic challenge in this case was the histological overlap between reactive xanthogranulomatous inflammation and ECD. ECD is now recognized as a clonal histiocytic neoplasm, with most patients harboring MAPK pathway alterations and many carrying the *BRAF*^V600E^ mutation [4,5]. However, authoritative clinical and pathological reviews emphasize that ECD is typically a multisystem disease, and its diagnosis requires not only appropriate histological and immunophenotypic findings but also a compatible pattern of clinical and radiological involvement, commonly affecting the skeleton, retroperitoneum, cardiovascular system, and lungs, ideally supported by molecular confirmation [15,16]. The mere presence of foamy histiocytes is insufficient for a diagnosis of ECD, as similar cells may be encountered in a variety of reactive inflammatory conditions, including xanthogranulomatous inflammation. In our patient, the lesion was confined to the rectovesical compartment and embedded within a chronic inflammatory milieu, without evidence of systemic involvement. This constellation strongly favored a reactive rather than clonal histiocytic process. This report has several important limitations. First, it is based on a single clinical case without a control group. Second, the relatively short follow-up period (approximately six months) limits conclusions regarding the durability of the response. Third, the absence of comparative data precludes any inference regarding the superiority of this therapeutic approach. Therefore, the findings should be considered hypothesis-generating.

### 6.2. Imaging and Differential Diagnosis

From an imaging standpoint, one of the most relevant challenges is the inability to reliably distinguish xanthogranulomatous inflammation from malignancy using conventional radiological techniques alone. Cross-sectional imaging typically demonstrates irregular masses with variable contrast enhancement, apparent infiltration of adjacent structures, and increased metabolic activity on PET–CT, all features that are commonly associated with malignant disease. Diffusion-weighted MRI may further reinforce this suspicion due to increased cellularity. These overlapping features frequently lead to extensive oncological work-up and may prompt consideration of radical surgical strategies in the absence of a definitive tissue diagnosis.

Another key differential diagnosis in this context is IgG4-related disease, which represents one of the main non-neoplastic conditions mimicking malignancy in the retroperitoneal and pelvic regions. Both IgG4-RD and xanthogranulomatous inflammation may present as mass-forming lesions with infiltrative features, heterogeneous enhancement, and potential involvement of adjacent structures, making radiological differentiation particularly challenging. However, important distinctions exist. IgG4-RD is typically characterized by a dense lymphoplasmacytic infiltrate, storiform fibrosis, and increased numbers of IgG4-positive plasma cells, often in the context of systemic disease involving multiple organs. In contrast, xanthogranulomatous inflammation is dominated by lipid-laden histiocytes within a chronic inflammatory background and is generally considered a localized reactive process [1,2,8]. In this regard, tissue biopsy remains the cornerstone for accurate diagnosis and for avoiding unnecessary radical interventions.

### 6.3. Therapeutic Considerations and Clinical Implications

Management in this case was primarily driven by anatomical considerations and the anticipated morbidity of surgery, as definitive resection would have required radical cystectomy with urinary diversion. Although xanthogranulomatous inflammatory masses are often managed surgically due to diagnostic uncertainty, this approach may be associated with considerable morbidity, particularly in anatomically complex regions such as the pelvis.

In this context, the present case supports a growing paradigm shift toward conservative treatment strategies in selected patients. Corticosteroids were used to suppress the inflammatory component, while everolimus was selected as an adjunctive immunomodulatory agent targeting mTOR-dependent pathways involved in histiocytic proliferation and immune activation. The decision to initiate mTOR inhibition was based on the histiocytic nature of the lesion and on therapeutic analogies with Erdheim-Chester disease, in which mTOR pathway targeting has shown clinical benefit [17,18,19].

This observation should be considered hypothesis-generating, and no conclusions regarding general therapeutic applicability can be drawn from a single case.

This finding is particularly relevant in clinical scenarios where radical surgery would entail significant functional impairment or morbidity.

Finally, this case underscores the importance of accurate tissue diagnosis and multidisciplinary decision-making in preventing overtreatment and guiding appropriate follow-up. When imaging findings are highly suspicious for malignancy but histology supports a benign inflammatory process, a conservative approach should be carefully considered. Prospective studies are needed to better define the role of targeted medical therapies in xanthogranulomatous inflammation and to identify patients who may benefit from non-surgical management strategies.

## 7. Conclusions

This case highlights the diagnostic and therapeutic challenges posed by xanthogranulomatous inflammation presenting as a deep pelvic mass with radiological features highly suggestive of malignancy. Beyond the individual clinical observation, it underscores key aspects of this condition, including its pathophysiological basis, its potential to mimic malignant disease on imaging, and the central role of histopathological confirmation. Importantly, our findings support the consideration of conservative medical management in selected patients, particularly when surgical intervention would be associated with high morbidity. A multidisciplinary approach and careful integration of clinical, radiological, and pathological data remain essential to guide optimal management.

## Data Availability

All data generated or analysed in this study are included in this published article. Additional details may be provided by the corresponding author upon reasonable request.
